# A Study in Involution

**Published:** 1899-10

**Authors:** A. Ferree Witmer

**Affiliations:** Philadelphia, Pa.


### Selections.

### A STUDY IN INVOLUTION.

By A. FERREE WITMER, M. D., Philadelphia, Pa.

[Proceedings of the Pathological Society of Philadelphia, Vol. II., No. 4.]

IT IS quite well known that man at different periods of his life is subject to varying forms of disease; thus we find disorders incident to childhood, those induced by the exposures of mature life, and those due to senility; in short, disorders of evolution, of maturity and of involution.

The time in which the first are observed is naturally from conception to the end of the period of growth, which occurs usually at the age of twenty; the second are found from that time to about the age of fifty, and the third from about fifty to the close of life.

The disorders most common to old age are fatty degeneration and calcification of the arteries—*i, e.*, we find at a time past the middle life abortive attempts on the part of nature to reconstruct the tissues, attempts that, owing to failing metabolism, never reach full organic requirement. The time at which this condition of enfeeblement may set in is not absolutely fixed; not infrequently it is found at thirty or even less, so that there has grown up in medicine the now trite saying that "man is as old as his arteries."

It is, however, to a broader conception of involution that I ask your attention: to what, indeed, might well be called the atavistic tendency of diseased tissue. As the nervous system is most highly specialised it is naturally to this tissue that one turns for confirmation of this view. Let us glance first at one of the most common of all the disorders of the nervous system—neurasthenia.

Two forms of this affection are recognised: the one called symptomatic, resulting from fatigue states, in which the nerve exhaustion is obviously secondary; the other, called essential, resulting from no known cause, unless one be willing to accept the possibility that these patients are—to use a vulgarism—born tired. It is significant that the essential form of neurasthenia does not respond favorably to treatment.

Upon examination of the bodily fluids in these individuals we are impressed by the excessive quantity of uric acid secreted; so great, indeed, that one is almost forced to the opinion that the normal secretion in these patients is similar to that in animals below the mammals in the zoologic scale.

That neurasthenia, although called a functional disease, has a definite pathology is highly probable. I have but to refer to the classic experiments of Hodge[1] to recall to you the marked cellular changes induced by excessive activity in the nervous system of the pigeon, bee, etc.

The findings of Hodge, in brief, are as follows: *a.* For the nucleus: 1, marked decrease in size; 2, change from a smooth and rounded to an irregular outline; 3, loss of open reticular appearance, with darker stain. *b.* For cell-cytoplasms: 1, slight shrinkage in size with vacuolation of spinal ganglia, considerable shrinkage with enlargement of peri-cellular lymph spaces for cells of cerebrum and cerebellum; 2, lessened power to stain and to reduce osmic acid.

It may reasonably be inferred from these careful experiments of Hodge that similar changes might result from over-stimulation of the cell bodies in man.

Granting, then, that neurasthenia may be classified as a disease and not as a "neurosis," we find that certain races fail to meet the nervous outlay demanded by the changing requirements of civilisation. This is particularly marked in the negro and American races.

Since the Civil War the ratio of insanity among the freed blacks has materially increased. Tuberculosis and syphilis, both of which are regarded as diseases coincident with civilisation, have also increased greatly in this race. The decay of the American race is equally well known. Another instance is afforded by the Russian Hebrew who shortly after immigrating to America, begins to feel the stress under which he is laboring. I can safely say that 90 per cent. of the Russian Hebrews who apply to our nervous infirmaries for treatment are neurasthenic.

There is apparent, then, in neurasthenia of the essential type a diminished virility of the nervous system and a reversion of the type of secretion.

The next nervous affection to which I invite your attention is unmistakably organic, amyotrophic lateral sclerosis. The wasting of the hand, which begins first, and becomes most advanced in degree, presents the characters of the simian type. The curved group which limits the ball of the thumb is present, but owing to the wasting of the eminence is not so marked as in the normal hand of man. The longitudinal grooves in the palm are also deeper than in the normal condition; the thumb, in short, becomes markedly subordinate.

In disorders of the nervous system, resulting from cerebral hemorrhage, and the like, we find that as improvement sets in the flexor groups of muscles are the first to respond. Frequently this relative over-activity becomes so great that the hand assumes the position of partial flexion known as the claw-hand. This condition is not difficult to interpret if we accept the proposition that the extensors are innervated from the brain and the flexors from the cord. Some of you have doubtless seen instances of a similar flexor activity in members of the feebleminded class who, while unable to perform any other gymnastic feat, can hang for quite long periods of time by grasping some horizontal support. In the early days of life of the normal child a like condition has been noticed.

Again, among the feebleminded there occurs not infrequently a typical Mongolian cast of features, known as the Mongolian form of idiocy. While I do not wish to be understood as considering the Mongolian, a race inferior to the Caucasian, yet the appearance of a

Mongolian type as the descendent of a pure Caucasian stock must be considered atavistic.

In mental diseases we find that when hallucinations are present those of hearing are most common, while those of smell are comparatively unknown. Bucke[2] has recently referred to this, and adds: 1, that the longer a race has been in possession of a given faculty the more universal will that faculty be in the race; and, 2, conversely, the more recent is any given faculty the more easily it is lost. In the ontogenetic development of man we must not forget that hearing is the latest to develop and smell the earliest.

F. P. Henry, quoted by Harrison Allen,[3] has shown that in pernicious anemia the red blood cells approach those of the lower animals in many if not all of their characteristics, viz., in their number, size, shape and amount of hemoglobin they carry. The number of red blood cells is reduced to a degree that is normal in the cold-blooded animals; the corpuscles being increased in diameter, are occasionally nucleated and have a tendency to assume an oval outline. The type of blood resembles the ancestral shape, Henry argues, because the corpuscles have lost the shape which is characteristic of their high position in the zoologic scale.

A condition of much interest occasionally found in man is rumination. It is noted in the human ruminant that the regurgitation of the food begins usually half an hour after its ingestion, and recurs at regular intervals for about two hours. The food during this time tastes sweet, not at all like the sour material that is thrown off in digestive derangement.

Rumination in man is generally regarded as practically identical in its mechanism with that of the herbivora, although as Riesman[4] observes, we are still ignorant of its ultimate cause.

Recently a case has been reported in which direct inheritance has been traced—the grandfather and father of the patient both being afflicted with this affection. This case is reported by Runge,[5] who argues as a likely cause the development of the stomach by the laws of natural selection, by which the conditions of the animal are adopted to the environment. Sinkler[6] is opposed to this view on the ground that the connection between man and the ruminant with his four stomachs is too remote. To the writer this last objection seems hardly tenable, as the time-element cannot be considered with any definiteness in the evolution of man.

It is generally agreed that the power of self-control largely distinguishes man from the lower animals. The morale of the defective class is notoriously bad. The appetites run wanton, every wish being gratified at the earilest possible moment.

We find in epilepsy, for instance, that gluttony is the rule. Of 100 cases taken consecutively, I find the appetite markedly ravenous in eight, excessive in 75, average in 4, variable in 6, and poor in only 7.

In the foregoing illustrations are represented disorders both of a functional and of an organic nature, those in which a fundamental disorganisation has taken place and those in which a mere quality has been disturbed.

Other disorders that show atavistic tendencies might be mentioned, but sufficient, I think, have been given to indicate the part that atavism plays in inducing derangement of the human nervous system.

REFERENCES.

1. Hodge, C. F.: A microscopical study of changes due to functional activity in nerve cells. Journal of Morphology, November, 1892.

2. Bucke, R. M.: An address before the Psychological Section of the British Association for the Advancement of Science, held in Toronto, June, 1897.

3. Allen, Harrison: Morphology as a factor in the study of disease. Transactions of the Congress of American Physicians and Surgeons, 1894.

4. Riesman, D.: Journal of Nervous and Mental Diseases, June, 1895.

5. Bunge, E.: Boston Medical and Surgical Journal, 1895.

6. Sinkler, W.: Rumination in Man. Journal of the American Medical Association April 9, 1898.

